# Improving control of carbide-derived carbon microstructure by immobilization of a transition-metal catalyst within the shell of carbide/carbon core–shell structures

**DOI:** 10.3762/bjnano.10.41

**Published:** 2019-02-11

**Authors:** Teguh Ariyanto, Jan Glaesel, Andreas Kern, Gui-Rong Zhang, Bastian J M Etzold

**Affiliations:** 1 Department of Chemical Engineering, Faculty of Engineering, Universitas Gadjah Mada, 55281 Yogyakarta, Indonesia; 2 Lehrstuhl für Chemische Reaktionstechnik, Friedrich-Alexander-Universität Erlangen-Nürnberg, Egerlandstrasse 3, 91058 Erlangen, Germany; 3 Ernst-Berl-Institut für Technische und Makromolekulare Chemie, Technische Universität Darmstadt, Alarich-Weiss-Strasse 8, 64287 Darmstadt, Germany

**Keywords:** carbon shell, catalytic graphitization, graphitic carbon, pore structure, transition metal

## Abstract

Carbon materials for electrical energy devices, such as battery electrodes or fuel-cell catalysts, require the combination of the contradicting properties of graphitic microstructure and porosity. The usage of graphitization catalysts during the synthesis of carbide-derived carbon materials results in materials that combine the required properties, but controlling the microstructure during synthesis remains a challenge. In this work, the controllability of the synthesis route is enhanced by immobilizing the transition-metal graphitization catalyst on a porous carbon shell covering the carbide precursor prior to conversion of the carbide core to carbon. The catalyst loading was varied and the influence on the final material properties was characterized by using physisorption analysis with nitrogen as well as carbon dioxide, X-ray diffraction, temperature-programmed oxidation (TPO), Raman spectroscopy, SEM and TEM. The results showed that this improved route allows one to greatly vary the crystallinity and pore structure of the resulting carbide-derived carbon materials. In this sense, the content of graphitic carbon could be varied from 10–90 wt % as estimated from TPO measurements and resulting in a specific surface area ranging from 1500 to 300 m^2^·g^−1^.

## Introduction

Carbon is a versatile material that has been widely utilized in many applications such as adsorption [1–3], catalysis [4–5], catalyst support [6–8], molecular sieves [9–10] and energy storage [11–13], owing to its large specific surface area and distinct pore character. For applications in which electrical conductivity plays an important role, e.g., battery electrodes, fuel-cell catalysts or supercapacitors [14–16], it is necessary for carbon to not only show porosity but also to feature a graphitic structure. The reason is that graphitic carbon consists of crystalline sp^2^-hybridized fractions that induce high electron conductivity. Moreover, an enhanced crystallinity is favorable in terms of chemical stability, which is required especially when working under harsh conditions.

Many synthetic approaches were employed to produce carbon combining porosity and graphitic structure [17–19]. Among them, the carbide-derived carbon (CDC) is a promising route. CDC can be synthesized through the selective extraction of metals or metalloid atoms from metal carbides (Me*_x_*C, e.g., TiC, SiC, VC, and Mo_2_C) by using halogen gases at elevated temperatures. Depending on the carbide and parameters employed during the synthesis, CDC can be varied from extremely amorphous to highly crystalline microstructures and from ultramicro- to mesoporous pore structures. Therefore, CDC is known as material with tunable microstructure and pore structures [20].

To produce CDC with a high content of graphitic structure, there are two possibilities that can be applied (neglecting a post-synthesis treatment after CDC synthesis). The first is very high synthesis temperatures (ca. 1500 °C) [4], which is, however, associated with a pronounced energy consumption for the reactor heating as well as with challenges to handle chlorine at such high temperatures. The second approach is using catalytic graphitization during the material synthesis. It typically requires only moderate temperatures (typically starting from 800 °C, depending on types of carbides [19]). Commonly used graphitization catalysts are transitions metals such as Fe, Ni, and Co [18,21–22]. The conventional method for catalytic graphitization is to mix the non-porous carbide and metal catalyst precursor prior to the selective etching at high temperature. Indeed, the graphitic content is present, but the overall material is rather inhomogeneous [18,23]. Most likely the physical powder mixture or the simple dip coating of the powder carbide precursor with the transition-metal catalyst lead to a very inhomogeneous starting mixture, which is responsible for the final heterogeneous combination. Immobilizing the transition metal-catalyst at each particle would ensure a homogeneous catalytic graphitization of the whole powder samples.

**Figure 1:**
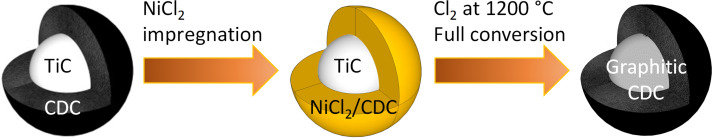
The schematic of graphitic CDC production via immobilization of transition-metal graphitization catalyst on CDC/carbide core–shell precursors.

We recently introduced the possibility to obtain core–shell particles in which a nanoporous carbon shell is covering a carbide core [14–15,24–25]. The porosity of this shell could suit for the immobilization of the transition-metal catalyst, as capillary forces would suck the impregnation solution within the shell and only excess solution would go into the voids between the particles. Subsequent chlorination of the carbide core to obtain carbide-derived carbon would be influenced by the transition-metal catalyst in the shell of each particle. This work studies the use of core–shell carbon/carbide hybrids to immobilize different amounts of graphitization catalyst as illustrated in Figure 1. The resulting microstructure and pore structure of the carbon material is characterized by X-ray diffraction (XRD), temperature-programmed oxidation (TPO) and physisorption analysis.

## Results and Discussion

### Properties of CDC-shell/carbide core starting material

**Figure 2:**
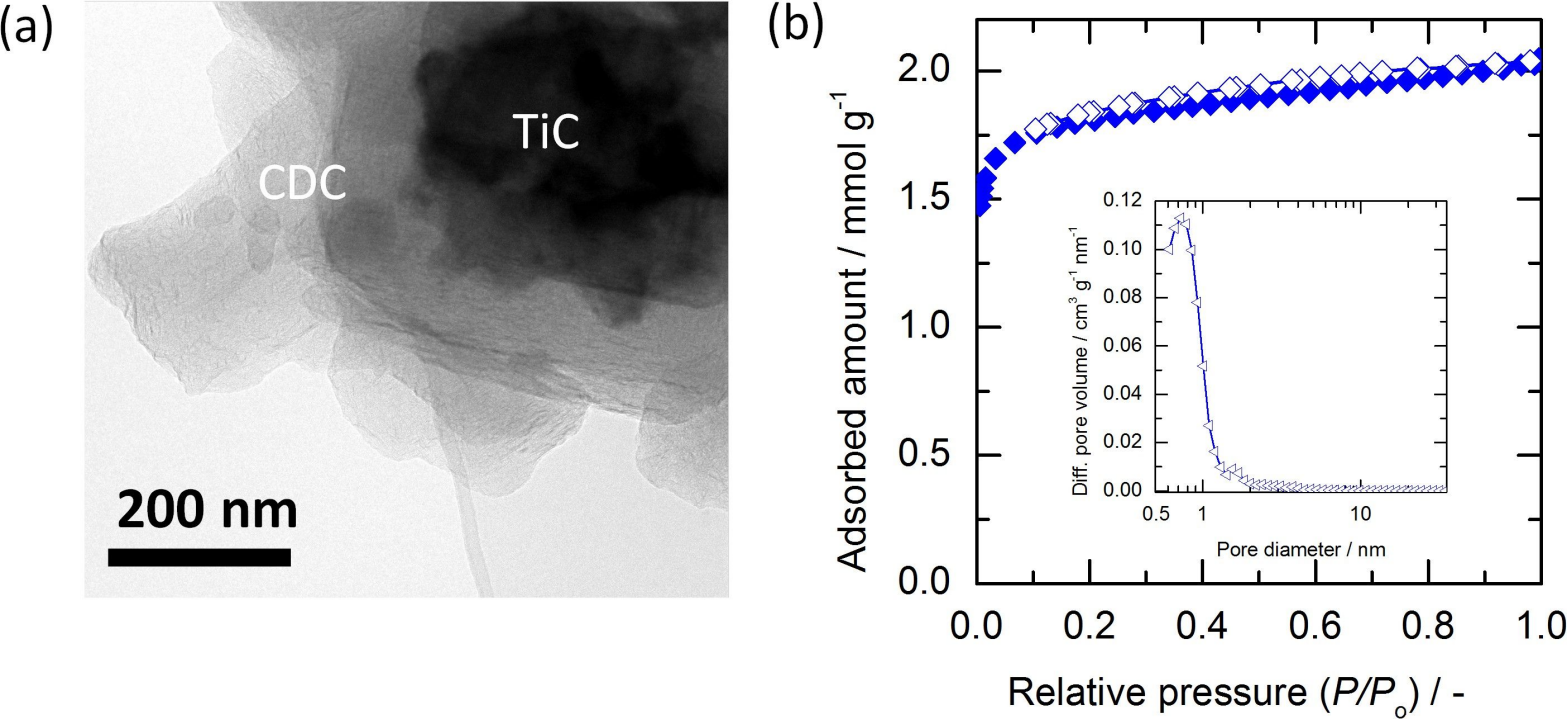
(a) TEM analysis of partially chlorinated carbide (CDC-shell) showing transparent CDC covering the carbide core; (b) N_2_-sorption isotherm of the CDC-shell and inset of its QSDFT pore size distribution.

First of all, the properties of the hybrid starting material (Figure 1, left) were studied. A partial conversion to obtain 30% shell and 70% core was set and confirmed by the mass loss recorded. Figure 2a shows a TEM image where clearly a porous carbon shell covering a carbide core is seen, which originates from the shrinking core like conversion mechanism in combination with the partial conversion [15,26].

Figure 2b shows the pore structure of the partially converted carbide at 800 °C characterized by N_2_ sorption analysis. The adsorption–desorption curve shows a similar shape compared to a typical fully CDC material synthesized at 800 °C but features a lower uptake due to the mass of the non-porous carbide core [15]. According to the IUPAC classification, the isotherm can be classified as type Ia suggesting a highly (ultra-)microporous material [27]. The pore size distribution (PSD) of the material was evaluated by using the quenched solid density functional theory (QSDFT) method (result displayed as inset in Figure 2b). CDC-shell contains mainly micropores (95 vol %) with a high peak of differential pore volume centered at ca. 0.8 nm. The surface area of CDC-shell is 160 m^2^·g^−1^ per total mass of material (shell and core). It can be concluded from the pore analysis that the porous CDC was obtained by the partial chlorination of carbide. For more details, the pore textural parameters are summarized below in Table 1.

To study whether the shell of partially converted carbide influences the distribution of the nickel precursor after the impregnation step, the impregnation of untreated titanium carbide was compared with the same loading (30 mg of nickel per gram of equivalent carbide). EDX mapping (see Figure S1 in Supporting Information File 1) of the impregnated core–shell material shows clearly the remaining core in the Ti K edge signal, while the Ni K and Cl K edges show that nickel chloride is homogeneously immobilized within the shell. A clustering on top of the particle seems not to take place. In contrast, the SEM image of the untreated titanium carbide shows nickel chloride crystals covering the particles. This is further corroborated through the EDS mapping (Figure S2 in Supporting Information File 1).

### Influence of nickel loading on the microstructure of the final carbon material

The porous-carbon-on-carbide-core material (CDC-shell) was impregnated with different amounts of nickel chloride hexahydrate (Figure 1, middle) and further chlorinated at 1200 °C to obtain the final material (Figure 1, right). The amount of nickel added was varied from 5 up to 60 mg of nickel per gram of equivalent carbide. The effect of nickel catalyst on the microstructure of final carbon was investigated using XRD, temperature-programmed oxidation (TPO), HRTEM and Raman spectroscopy.

**Figure 3:**
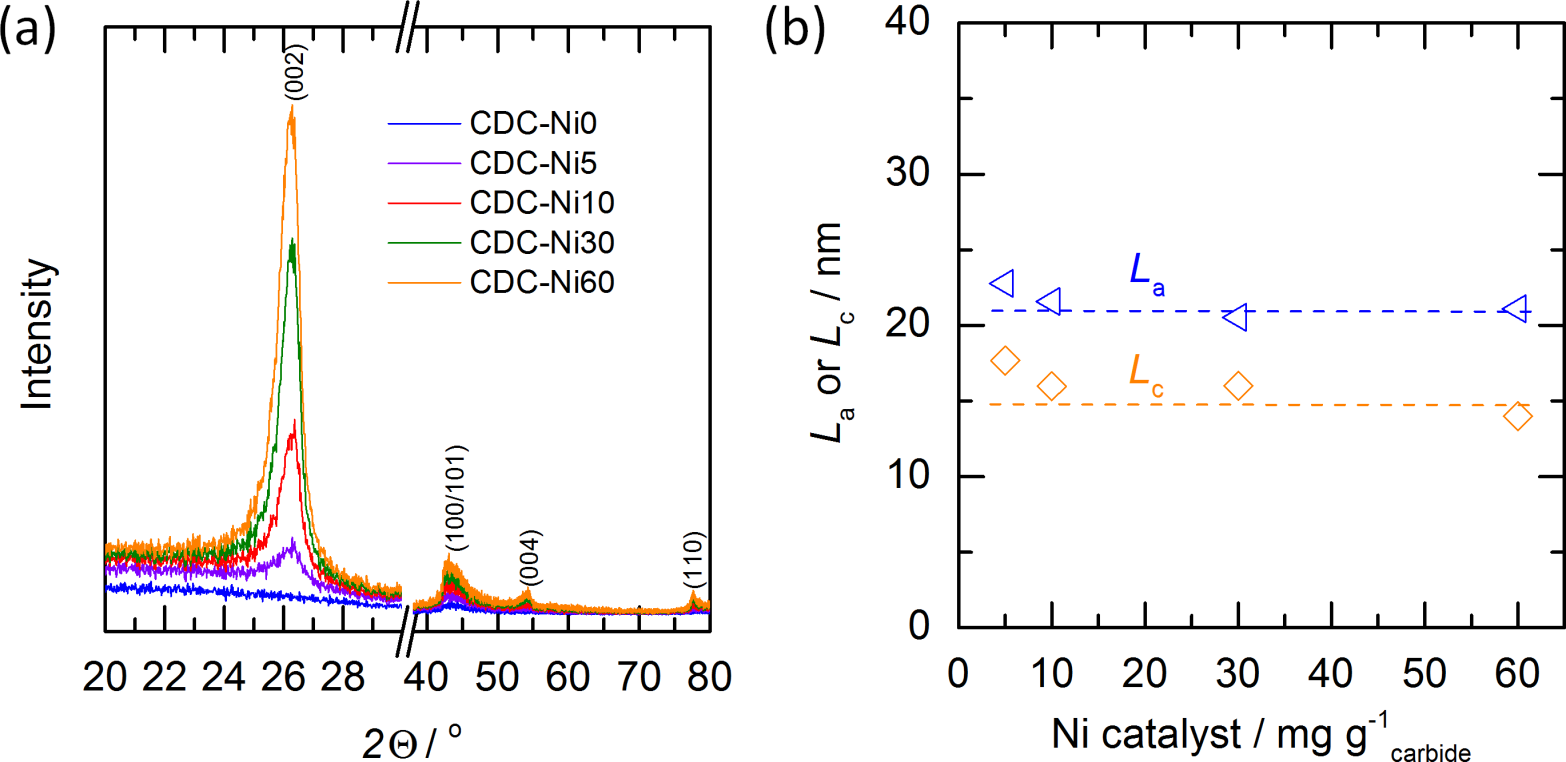
(a) XRD pattern and (b) the crystallite dimension for the in-plane (*L*_a_) and cross section of multi-layer carbons (*L*_c_) of final CDCs.

The XRD patterns for the different catalyst loadings are given in Figure 3a. The CDC-Ni0 reference material shows no reflexes indicating an amorphous character, which is in agreement with the literature [15]. Once adding graphitization catalyst (CDC-Ni5 to CDC-Ni60) clearly graphitic reflexes of C(002) and C(100/101) at 2θ ≈ 26° and 2θ ≈ 43° and even of C(004) and C(110) are observable. Figure 3a also depicts that the diffraction peak intensity increases with higher nickel loading, indicating a larger portion of crystalline carbon with rising nickel content. To investigate further the effect of nickel loading to the crystallite dimension, the parallel and in-plane symmetry crystallite sizes corresponding to C(002) (2θ ≈ 26°) and C(100) (2θ ≈ 43°) were evaluated using the Scherrer equation (peak deconvolution, see Experimental section). It is noted that the Scherrer equation provides an only rough estimation of crystal dimensions but can serve as basis for the discussion of microstructural trends. The evaluation reveals that the crystallite dimensions, i.e., width (*L*_a_) and height (*L*_c_) for final CDC are largely independent of the amount of employed nickel catalyst (see Figure 3b). Despite the relative constant crystal sizes, the increasing intensity of the XRD reflexes indicates that the amount of crystalline carbon compared to amorphous phase is increasing with higher nickel loading.

**Figure 4:**
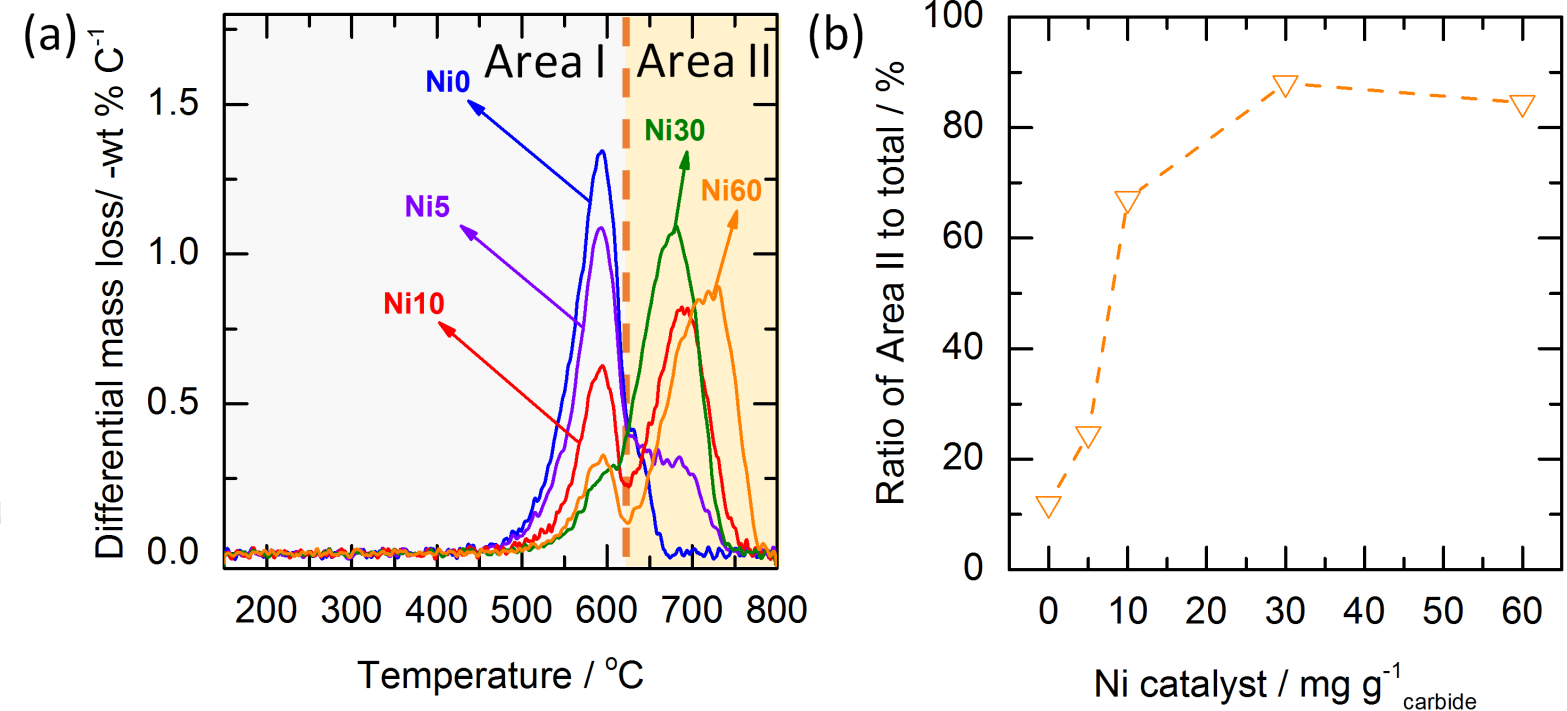
(a) Temperature-programmed oxidation profile of final CDC; (b) the calculated fraction of Area II representing content of more graphitic carbon.

TPO was employed to probe the ratio between of amorphous and crystalline carbon, based on the different oxidation stability [4,28]. Differential mass-loss curves from the TPO analysis of materials with varying nickel loading are displayed in Figure 4a. It can be seen that CDC-Ni0 shows only a single oxidation peak with a maximum at approx. 596 °C. The CDC-Ni5 reference with the smallest amount of graphitization catalyst added, exhibits also a large signal with an oxidation peak of 595 °C and shows a second peak rising at approx. 700 °C. With even higher nickel contents (CDC-Ni10 and higher) clearly two oxidation peaks can be distinguished, where the first peak corresponds to the more amorphous carbon obtained without adding catalyst (CDC-Ni0). It can therefore be speculated that the second peak at higher oxidation temperatures belongs to the graphitic domains generated with the nickel catalyst.

As there is a distinct separation of both peaks at 610 °C, the TPO signal is divided into two areas, i.e., “Area I” in the region below 610 °C and “Area II” in the region above 610 °C. From integrating both regions the ratio between amorphous and graphitic carbon can be roughly estimated. Figure 4b plots this ratio as a function of the Ni catalyst loading. It can be clearly seen that the portion of crystalline carbon increases to 67% when adding 5 and 10 mg of nickel per g of carbide. Adding more nickel (30 mg·g^−1^) increases the ratio up to approx. 90% while a further increase to 60 mg·g^−1^ shows no additional improvement.

**Figure 5:**
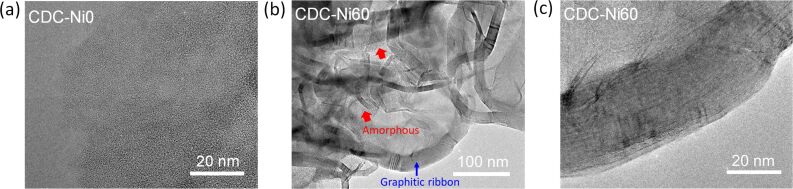
TEM images of CDC-Ni0 (a) and CDC-Ni60 (b,c).

TEM images of crushed particles of CDC-Ni0 and CDC-Ni60 are given in Figure 5 and support the findings. Clearly CDC-Ni0 exhibits an amorphous character. The CDC-Ni60 exhibits graphitic character indicated by the formation of graphitic ribbons (Figure 5b). The stacking height corroborates the XRD diffractogram evaluation. Parallel fringes are seen in the magnification of the graphitic ribbons (Figure 5c). From the TEM study it seems that also for CDC-Ni60 some amorphous carbon is homogeneously distributed among the graphitic domains (Figure 5b). Raman spectra were recorded for CDC-Ni0, CDC-Ni10 and CDC-Ni60 and are given in Supporting Information File 1 (Figure S3). Surprisingly, in contrast to TPO, XRD and TEM, no strong differences in crystallinity of the samples can be observed by using Raman spectroscopy. All spectra are characterized by the presence of two more or less overlapping D- and G-bands centered at ca. 1325 and 1583 cm^−1^. CDC-Ni0 shows a slightly higher level of disorder, while the spectra of CDC-Ni10 and CDC-Ni60 are similar. The reason for the deviation from the other characterization results could be the penetration depth of the Raman laser, which probably is probing especially the shell of the core–shell material. As the initial shell is produced without graphitization catalyst here, more amorphous carbon is expected. The Raman results indicate, that the initial amorphous shell is not strongly recrystallizing during the second chlorination step. This could be also the reason for the amorphous carbon detected in the TPO characterization even for high nickel loadings.

The results show that the loading of graphitization catalyst allows one to tune the content of crystalline carbon. Furthermore, if sufficient catalyst is immobilized within the porous shell, the whole particles seem to benefit from the graphitization catalyst. It also needs to be noted that in the final material no remaining nickel was found by XRD (Figure 3a), in the ash of the TPO measurements (see Figure S4 in Supporting Information File 1) or in the TEM images. The absence of nickel residues can be explained by the formation of volatile NiCl_2_ during the chlorination of the core at 1200 °C [29].

### Influence of nickel loading on the pore structure of the final carbon material

**Figure 6:**
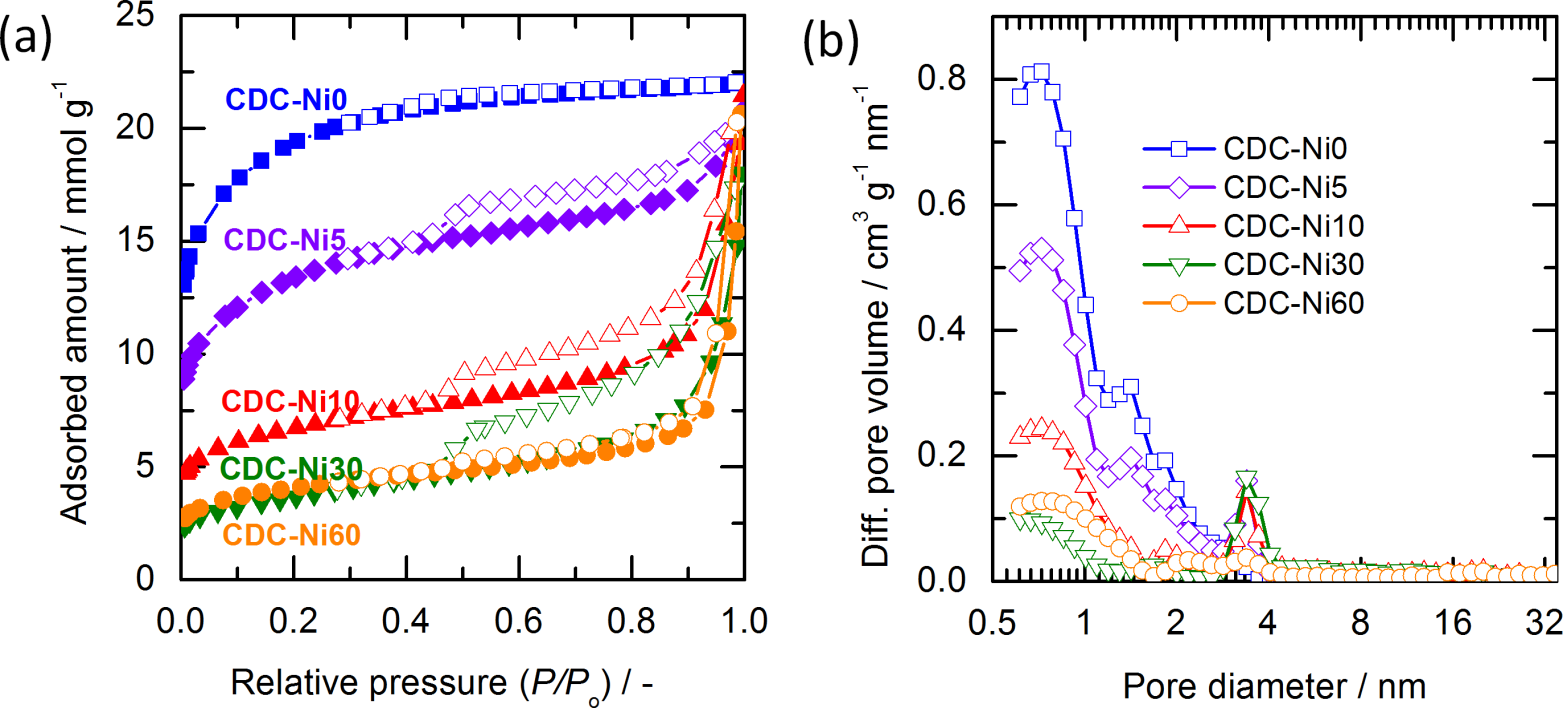
(a) N_2_-sorption isotherm of final CDC material (closed and open symbols show the adsorption and desorption branches, respectively); (b) Pore size distribution of final CDC evaluated by QSDFT method.

Figure 6a shows the resulting nitrogen-sorption isotherms for varying amounts of nickel loading. The material without graphitization catalyst (CDC-Ni0) shows an isotherm with a wider knee in the low-pressure range (type Ib) isotherm, indicating a broad range of micropores. This is in accordance with pore size distributions obtained for TiC-CDC at 1200 °C [4,30]. The addition of 5 mg of nickel per gram of carbide (CDC-Ni5) already shows a pronounced influence on the resulting isotherm, which is a combination of type I and type II with a pronounced H3 hysteresis loop. It suggests that a larger pore exists in CDC-Ni5, which is likely induced by the graphitizing effect of the nickel catalyst, as described in [18,31]. Increasing the nickel loading from 5 to 30 mg·g^−1^ carbide, leads to similar isotherm shapes but a decrease in the adsorbed volume of N_2_ in the low-pressure range. The pore size distributions evaluated by the QSDFT model are displayed in Figure 6b. CDC-Ni0 displays mainly pores in the micropore regime (<2 nm). On the other hand, the CDCs produced with the aid of the nickel catalyst show pores in the range of 3–4 nm, which are not present in the CDC-Ni0 sample.

The structural properties (specific surface area (SSA), total pore volume (*V*_T_) and mean pore size (*d*_m_)) are summarized in Table 1. There are two types of SSA, SSA_N2_ and SSA_CO2_, obtained from nitrogen and carbon dioxide sorption analysis. Due to the low pressure of the CO_2_ analysis (*P*/*P*_0_ = 2.9 × 10^−2^), only pores in the micropore regime up to 1.5 nm can be probed [32]. Therefore, the mesopore/macropore structures can be roughly estimated by subtracting the contribution of micropore structures (CO_2_ sorption) from the total pore structures (N_2_ sorption). The reference of CDC-Ni0 features SSA_N2_ of ca. 1500 m^2^·g^−1^ and a mean pore size of 0.94 nm. CDC-Ni5 displays a lower surface area of ca. 1000 m^2^·g^−1^, which is caused by the presence of larger pores as a consequence of the nickel catalyst. The mean pore size of CDC-Ni5 increases by 32% compared to the CDC-Ni0. The specific surface area follows a reverse trend with respect to the nickel content (up to 30 mg·g^−1^ carbide), but the average pore size increases, e.g., it is 3.53 nm for CDC-Ni30 and therefore more than three times as large as that of pristine CDC-Ni0. The results of CO_2_ sorption analysis corroborate the finding that the micropore portion decreases from 86 to 13 vol % when employing nickel loadings of 0–30 mg·g^−1^ carbide. Interestingly, increasing the nickel loading from 30 to 60 mg·g^−1^ does not lead to strong changes in the pore structure. In accordance with the TPO results, where the ratio of amorphous to graphitic carbon also did not change further, it can be speculated that 30 mg·g^−1^ of nickel are the maximum amount of catalyst needed for full graphitization.

**Table 1:** Structural parameters of CDC material characterized by N_2_- and CO_2_-sorption.

sample	SSA_N2_^a^	*V*_T_	*d*_m_^b^	SSA_CO2_^c^	*V*_CO2_^d^	*V*_CO2_/*V*_T_
	[m^2^/g]	[cm^3^/g]	[nm]	[m^2^/g]	[cm^3^/g]	[%]

CDC-shell	160	0.07	0.81	157	0.06	86
CDC-Ni0	1494	0.71	0.94	1278	0.47	66
CDC-Ni5	1030	0.64	1.24	785	0.29	45
CDC-Ni10	533	0.60	2.24	423	0.16	27
CDC-Ni30	297	0.52	3.53	176	0.07	13
CDC-Ni60	309	0.45	2.92	258	0.10	22

^a^Specific surface area obtained by N_2_-sorption analysis, ^b^mean slit-pore size, (*d*_m_) = 2*V*_T_/SSA_N2_, ^c^specific surface area obtained by CO_2_ sorption analysis, ^d^pore volume taken from CO_2_ sorption analysis.

## Conclusion

A new synthesis strategy to obtain graphitic CDC was introduced in which nickel as graphitization catalyst is immobilized on a porous shell covering each particle. This approach allows one to vary the ratio of graphitic to amorphous carbon in the final material through the amount of immobilized nickel. Increasing the loading up to 30 mg_Ni_·g^−1^_carbide_ increased the graphitic content from 10 to 90% as estimated from TPO measurements, while the crystalline character (stacking height and width) is independent of the graphitic portion. This has a direct influence on the resulting pore structure showing a decreasing amount of micropores and increasing amount of meso- and macropores. Increasing the nickel loading above 30 mg_Ni_·g^−1^_carbide_ did not change the material properties further and probably additional nickel can be seen to some extent as inert material not participating in the conversion. The new synthesis route seems to result in more homogeneous materials and allows for a better control of the final material properties.

## Experimental

### Materials

Commercial TiC (*d*_ave_ of 90 µm, 99.8%, Goodfellow) was employed as carbon precursor. Chlorine (purity 2.8, Linde AG) and hydrogen (purity 5.0, Linde AG) diluted by helium (purity 4.6, Linde AG) were used to perform reactive extraction of carbide (chlorination) and subsequent carbon surface annealing. Nickel chloride hexahydrate (99.95% purity, Alfa Aesar) was used as precursor of the Ni catalyst.

### Synthesis of carbon shell/carbide core starting material

The synthesis of hybrid particles where carbide cores are covered with a porous carbon shell, was reported previously in detail [15]. Briefly, a vertical quartz tube reactor (*d*_i_ = 0.034 m, *l* = 1 m) was employed to perform partial chlorination of carbide to carbon. About 1 g of TiC powder was loaded on the top of a quartz frit of a quartz tube. The reactor was then placed in an isothermal zone of the vertical furnace (Gero Company, Germany). After the tightness of reactor was verified, the reactor was heated to 800 °C under helium flow (superficial velocity, *v* = 0.015 m·s^−1^) with a heating rate of 10 °C·min^−1^. The chlorination reaction was then carried out at 800 °C by dosing chlorine (0.5 mol·m^−3^ Cl_2_ diluted in helium, *v* = 0.015 m·s^−1^) for 30 min reaction time. The chlorine flow was then stopped, and the reactor was flushed with helium. To remove residual chlorine in the pores, the sample was subsequently treated with 0.5 mol·m^−3^ hydrogen. Eventually, the reactor was cooled down to ambient temperature under helium purge. The carbon shell/carbide core intermediate produced is denominated as CDC-shell.

### Impregnation of nickel in carbon shell/carbide core starting material

The nickel precursor was loaded to CDC-shell through wet impregnation, i.e., about 1 g of CDC-shell was mixed with a defined amount of nickel chloride hexahydrate dissolved in 3 mL ethanol. The solution was homogenized by ultrasonication for 30 min. The solvent was evaporated, and the nickel chloride-loaded CDC-shell was dried in an oven at 60 °C overnight. The loading of nickel on CDC-shell (wt Ni/wt equivalent carbide) was set to 5, 10, 30, and 60 mg·g^−1^_carbide_. The equivalent mass of carbide (*m*_TiC_) is determined using Equation 1.

[1] $$m_{\text{TiC}}=\frac{m_{\text{CDC-shell}}}{\left(1-X\frac{M_{\text{Ti}}}{M_{\text{TiC}}}\right)}\;,$$

where *m*_CDC-shell_ is the mass of carbon shell/carbide core starting material, *X* is the conversion rate, and *M*_Ti_ and *M*_TiC_ are the molar weight of Ti and TiC, respectively.

### Synthesis of final carbide-derived carbon

The Ni/CDC-shell was further chlorinated at 1200 °C to complete the conversion of the carbide to the carbon. This reactive extraction was carried out using a horizontal chlorination setup as described in [4]. The reaction conditions were set to 3 cm·s^−1^ superficial velocity, 1 mol·m^−3^ chlorine and 3 h reaction time. To remove residual chlorine, an annealing treatment using hydrogen (0.5 mol·m^−3^) again was carried out after the extractive reaction. The nomenclature of the final carbon material obtained is “CDC” followed by “Ni” and catalyst loading. For instance, CDC-Ni30 refers to the CDC material prepared by i) impregnation of CDC-shell with NiCl_2_·6H_2_O with 30 mg Ni·g^−1^_CDC-shell_ and ii) chlorination until full conversion of Ni/CDC-shell at 1200 °C.

### Characterization methods

**Figure 7:**
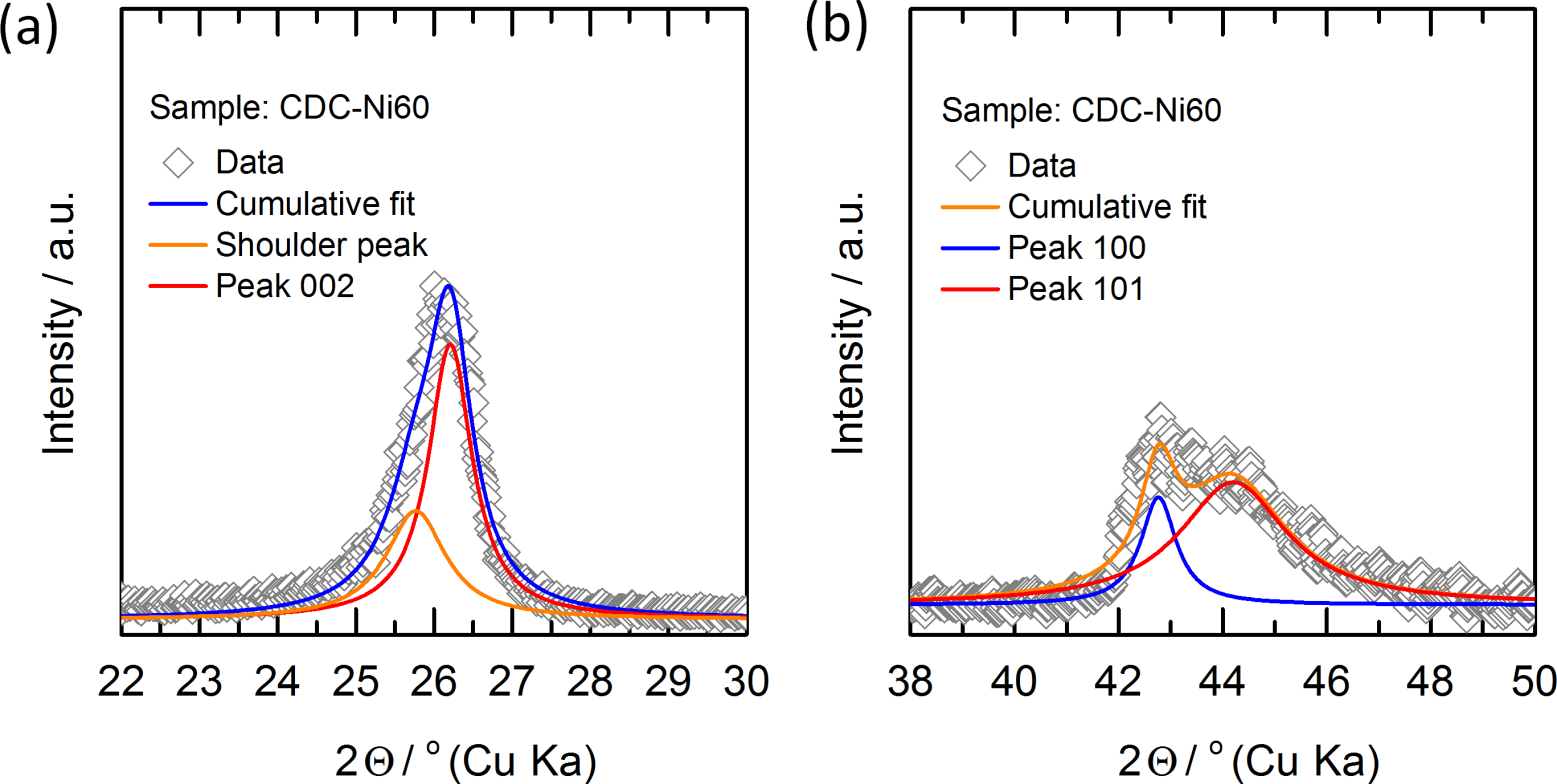
Examples of peak deconvolutions of XRD diffractograms at (a) C(002) and (b) C(100/101).

The pore structure of CDC-shell and final CDC materials was characterized by N_2_ sorption at −196 °C using liquid nitrogen as coolant (Quantachrome Quadrasorb Si-MP) and CO_2_ sorption measurements at 0 °C using a cryostat (Quantachrome Nova 4200e). Before the measurement, the sample was degassed at 250 °C for 4 h (N_2_ sorption) or 100 °C for 24 h (CO_2_ sorption). The pore size distributions were evaluated from the sorption-isotherm data by using quenched solid density functional theory (QSDFT) equilibrium models for carbon with slit-shaped pores [33] provided by the QuadraWin 5.04 software (Quantachrome Instruments, USA). Temperature-programmed oxidation measurements (TPO) of the carbon materials were recorded in a Netzsch STA 409 PC Luxx (Germany) under air flow. The method consisted of isothermal drying at 150 °C for 1 h followed by heating from 150 to 800 °C at a constant ramp rate of 2.5 °C·min^−1^. Peak deconvolution of the TPO curves was carried out with two bi-Gaussian functions. Raman spectra were taken using Jobin Yvon HR 800 with a HeNe laser (633 nm and 20 mW power). Peak deconvolution of Raman spectra was carried by peak fitting with four Lorentzian/Gaussian functions as described in [15]. Energy-dispersive spectroscopy (EDS) measurements were performed on a scanning electron microscope (Philips XL30 FEG, 30 kV) equipped with an EDAX X-ray detector. Transmission electron microscopy (TEM) images were captured using a JEOL JEM-2100F microscope operated at 200 kV. The TEM samples were prepared by placing a drop of catalyst powder dispersion in deionized water onto a carbon-coated Au grid (G200F1, Quantifoil), followed by drying under ambient conditions. XRD patterns were recorded using a Philips X’pert Pro by PANalytical, Netherlands (40 kV and 40 mA using Cu Ka radiation). The XRD diffractograms were recorded in the 2θ range of 2–80° in steps of 0.03° with an acquisition time of 5 s per step. The XRD diffractograms were deconvoluted to evaluate the properties of graphitic reflexes (see exemplary deconvolution in Figure 7).

The graphite dimension (*L*_a_ of the in-plane and *L*_c_ of the cross section size) was evaluated by the Scherrer equation shown in Equation 2 [34].

[2] $$L_{\text{a/c}}=\frac{K\text{λ}}{\text{β}\cos\text{θ}}\;,$$

where λ is the X-ray wavelength (0.154 nm), θ is the diffraction angle, β is the full width at half maximum (FWHM) of the deconvoluted peak in radian units and *K* is a constant (*K* = 1.84 for *L*_a_ at C(100/101) and *K* = 0.89 for *L*_c_ at C(002)).

## Supporting Information

File 1 Additional data on SEM-EDX, Raman spectroscopy and temperature-programmed oxidation.

## References

[1] Kameda T, Ito S, Yoshioka T (2017). J Dispersion Sci Technol.

[2] Altenor S, Carene B, Emmanuel E, Lambert J, Ehrhardt J-J, Gaspard S (2009). J Hazard Mater.

[3] Yi H, Li F, Ning P, Tang X, Peng J, Li Y, Deng H (2013). Chem Eng J.

[4] Gläsel J, Diao J, Feng Z, Hilgart M, Wolker T, Su D S, Etzold B J M (2015). Chem Mater.

[5] Wang L, Yao Y, Zhang Z, Sun L, Lu W, Chen W, Chen H (2014). Chem Eng J.

[6] Faria P C C, Órfão J J M, Pereira M F R (2009). Appl Catal, B.

[7] Kirilin A V, Hasse B, Tokarev A V, Kustov L M, Baeva G N, Bragina G O, Stakheev A Y, Rautio A-R, Salmi T, Etzold B J M (2014). Catal Sci Technol.

[8] Munoz M, Zhang G-R, Etzold B J M (2017). Appl Catal, B.

[9] Prasetyo I, Rochmadi R, Wahyono E, Ariyanto T (2017). Eng J.

[10] Silvestre-Albero A, Rico-Francés S, Rodríguez-Reinoso F, Kern A M, Klumpp M, Etzold B J M, Silvestre-Albero J (2013). Carbon.

[11] Sevilla M, Foulston R, Mokaya R (2010). Energy Environ Sci.

[12] Prasetyo I, Rochmadi R, Ariyanto T, Yunanto R (2013). Indones J Chem.

[13] Gütlein S, Burkard C, Zeilinger J, Niedermaier M, Klumpp M, Kolb V, Jess A, Etzold B J M (2015). Environ Sci Technol.

[14] Zeiger M, Ariyanto T, Krüner B, Peter N J, Fleischmann S, Etzold B J M, Presser V (2016). J Mater Chem A.

[15] Ariyanto T, Dyatkin B, Zhang G-R, Kern A, Gogotsi Y, Etzold B J M (2015). Microporous Mesoporous Mater.

[16] Schlange A, dos Santos A R, Hasse B, Etzold B J M, Kunz U, Turek T (2012). J Power Sources.

[17] Jin H, Li J, Gao L, Chen F, Zhang H, Liu Q (2016). Int J Hydrogen Energy.

[18] Kormann M, Gerhard H, Zollfrank C, Scheel H, Popovska N (2009). Carbon.

[19] Sevilla M, Fuertes A B (2006). Carbon.

[20] Gogotsi Y, Nikitin A, Ye H, Zhou W, Fischer J E, Yi B, Foley H C, Barsoum M W (2003). Nat Mater.

[21] Jeong J-H, Bae H-T, Lim D-S (2010). Carbon.

[22] Borchardt L, Hasché F, Lohe M R, Oschatz M, Schmidt F, Kockrick E, Ziegler C, Lescouet T, Bachmatiuk A, Büchner B (2012). Carbon.

[23] Xu J, Zhang R, Ge S, Wang J, Liu Y, Chen P (2013). Mater Chem Phys.

[24] Ariyanto T, Kern A M, Etzold B J M, Zhang G-R (2017). Electrochem Commun.

[25] Ariyanto T, Zhang G-R, Riyahi F, Gläsel J, Etzold B J M (2017). Carbon.

[26] Knorr T, Kaiser M, Glenk F, Etzold B J M (2012). Chem Eng Sci.

[27] Thommes M, Kaneko K, Neimark A V, Olivier J P, Rodriguez-Reinoso F, Rouquerol J, Sing K S W (2015). Pure Appl Chem.

[28] Pérez C R, Yeon S-H, Ségalini J, Presser V, Taberna P-L, Simon P, Gogotsi Y (2013). Adv Funct Mater.

[29] Galetz M C, Rammer B, Schütze M (2015). Mater Corros.

[30] Presser V, Heon M, Gogotsi Y (2011). Adv Funct Mater.

[31] Leis J, Perkson A, Arulepp M, Nigu P, Svensson G (2002). Carbon.

[32] Schmirler M, Glenk F, Etzold B J M (2011). Carbon.

[33] Gor G Y, Thommes M, Cychosz K A, Neimark A V (2012). Carbon.

[34] Dinnebier R E, Billinge S J L (2008). Powder Diffraction.

