# Supplementary material for: Improving control of carbide-derived carbon microstructure by immobilization of a transition-metal catalyst within the shell of carbide/carbon core–shell structures
Source: Beilstein J Nanotechnol. 2019 Feb 11;10:419–27. doi: 10.3762/bjnano.10.41 (PMC6404475; doi:10.3762/bjnano.10.41)
Supplement: File 1 — Additional data on SEM-EDX, Raman spectroscopy and temperature-programmed oxidation. [file Beilstein_J_Nanotechnol-10-419-s001.pdf]

BEILSTEIN JOURNAL OF NANOTECHNOLOGY

Supporting Information

for

Improving control of carbide-derived carbon microstructure by immobilization of a transition-metal catalyst within the shell of carbide/carbon core–shell structures

Teguh Ariyanto, Jan Glaesel, Andreas Kern, Gui-Rong Zhang and Bastian J. M. Etzold

Beilstein J. Nanotechnol. **2019,** *10,* 419–427. doi:10.3762/bjnano.10.41

Additional data on SEM-EDX, Raman spectroscopy and temperature-programmed oxidation

License and Terms: This is a supporting information file under the terms of the Creative Commons Attribution License (http://creativecommons.org/licenses/by/4.0). Please note that the reuse, redistribution and reproduction in particular requires that the authors and source are credited.
The license is subject to the *Beilstein Journal of Nanotechnology* terms and conditions: (https://www.beilstein-journals.org/bjnano)

A. Additional data on SEM-EDX

Figure S1 displays SEM image and elemental mapping of material of nickel chloride/CDC-shell. Element of titanium is observed in the core of a particle, while Ni and Cl are selectively immobilized within the shell.

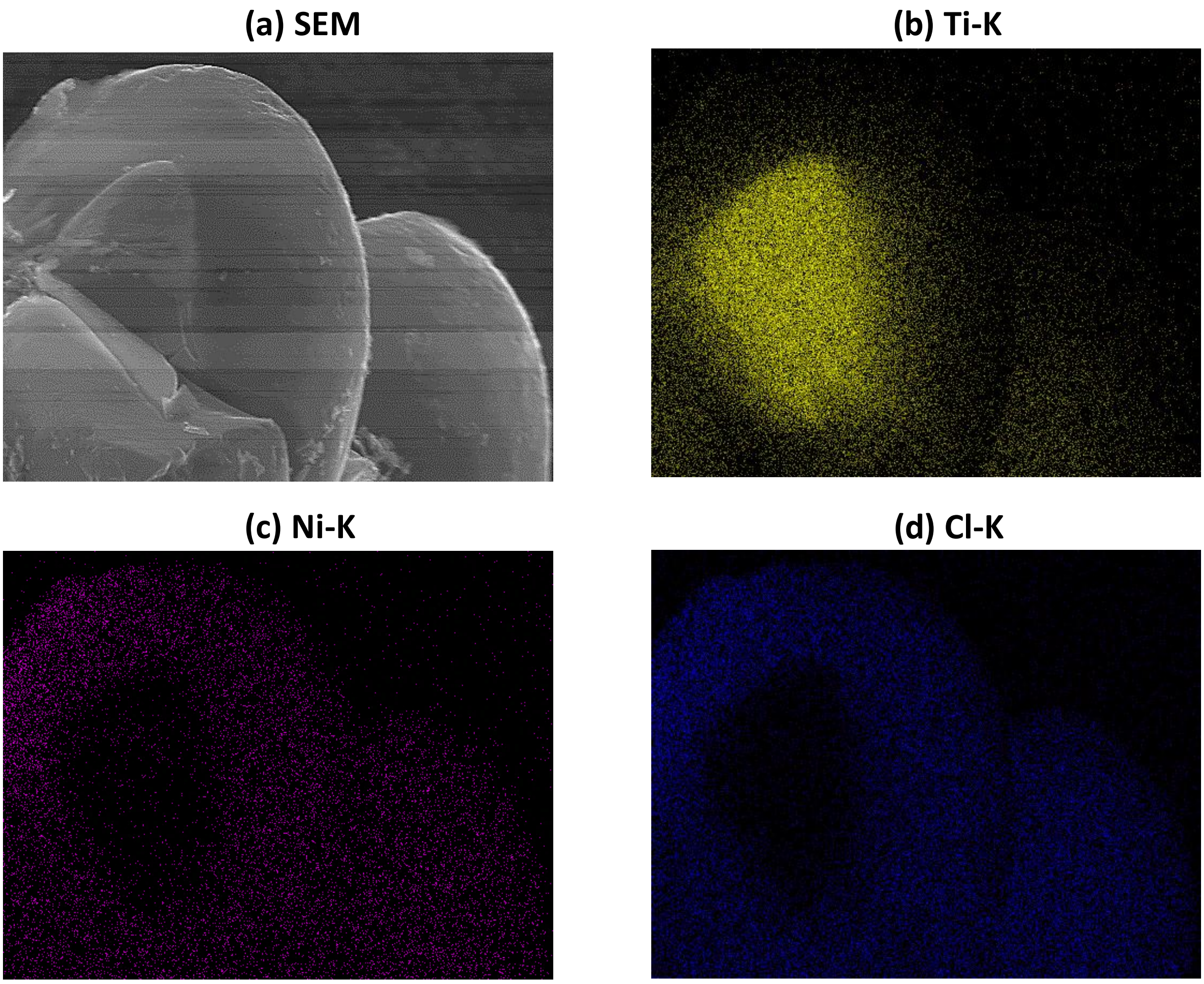

Figure S1: SEM image (a) and elemental mapping of Ti (b), Ni (c) and Cl (d) of nickel chloride/CDC-shell.

Figure S2 shows SEM image and elemental mapping of material of nickel chloride immobilized in untreated TiC particles. From SEM image (Figure S2a), it can be directly noticed that nickel chloride is immobilized in the outer surface of TiC particle. Elemental mappings of Ti, Ni and Cl confirm the SEM image.

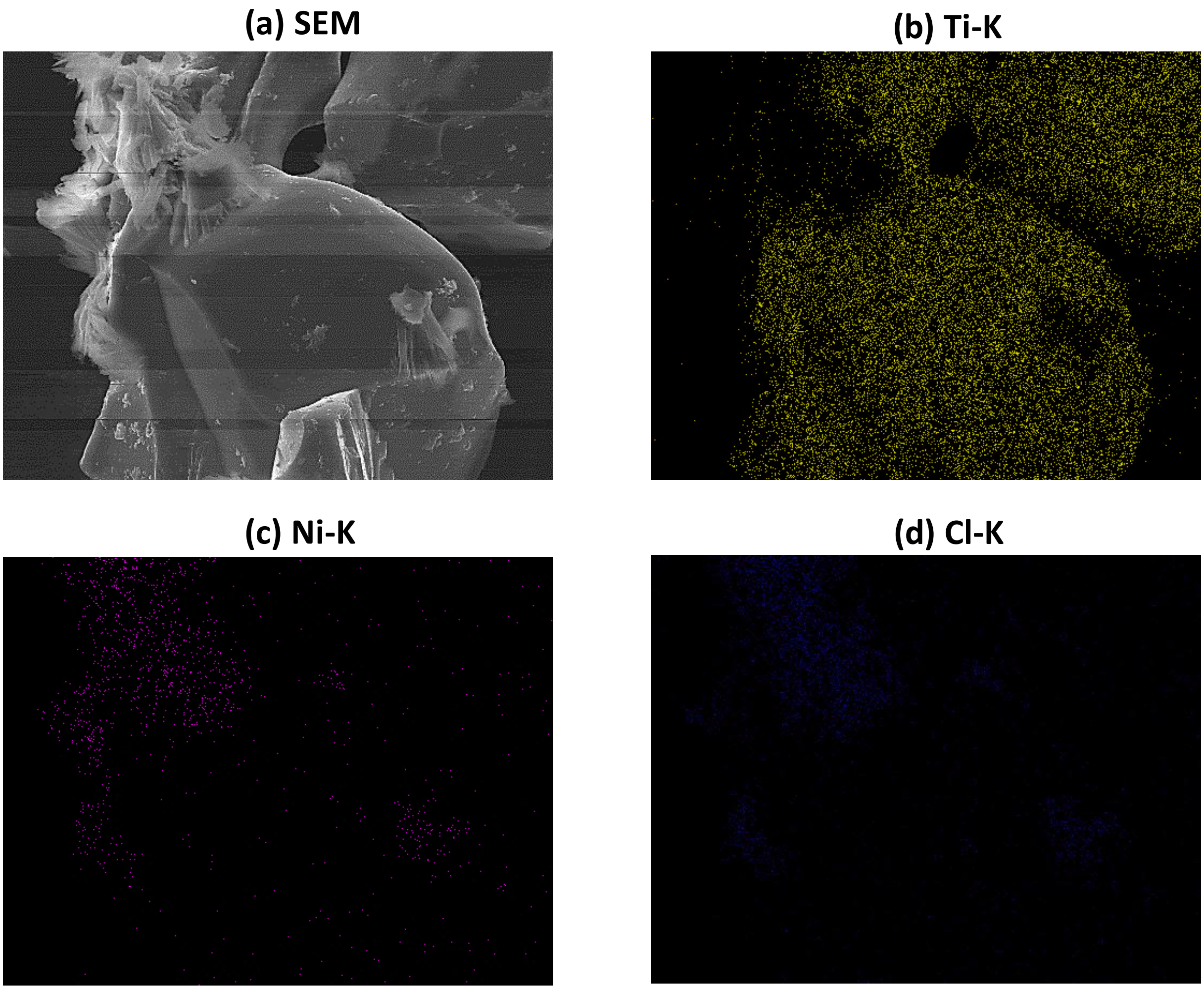

Figure S2: SEM image (a) and elemental mapping of Ti (b), Ni (c) and Cl (d) of nickel chloride/TiC.

B. Additional data on Raman spectroscopy

The Raman spectra and the results on peak deconvolution are shown in Figure S3 and Table S1. The peak properties were derived by applying peak deconvolution using four Laurentzian/Gaussian type peaks. The example of the fitting procedure is given in detail for CDC-Ni0. The graphitic (G) and the disorder-induced (D) bands are located at approx. 1320 and 1590 cm^{-1}, and the distinct shoulder peaks (D_2 at 1170 and G_2 at 1510 cm^{-1}) are typically observed features for activated carbons. Graphitic crystallites can be indicated by the intensity ratios of the D and G bands for different carbon microstructures [1]. CDC-Ni0, CDC-Ni10

and CDC-Ni60 shows a closely resemble structure as highlighted by the similarities in the FWHM and the intensity ratio.

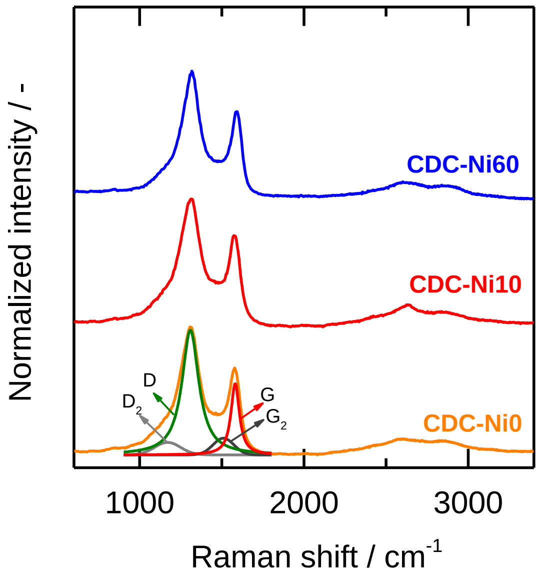

Figure S3: Raman spectra of final materials of CDC-Ni0, CDC-Ni10 and CDCNi-60.

Table S1: Peak deconvolution results of Raman spectra of CDC-Ni0, CDC-Ni10 and CDC-Ni60.

Material	Centre [cm^{-1}]		FWHM [cm^{-1}]		
	D1	G1	D1	G1	I_D/I_G
CDC-Ni60	1315	1592	123	59	1.58
CDC-Ni10	1308	1581	130	62	1.71
CDC-Ni0	1311	1582	138	62	1.73

C. Additional data on temperature-programmed oxidation (TPO)

Figure S4 shows curves of remaining mass of different materials during oxidation in air while heating with a constant temperature ramp. CDC-Ni0 displays one step decreasing curve indicating a homogeneous carbon structure. CDC-Ni5 till -Ni60 show a two-step decreasing curve and pronounced knee. This indicates that there is a mixture of carbon structures [2]. Moreover, increasing content of graphitization catalyst of nickel results in higher onset temperature of material. Even for the sample with the highest Ni loading (CDC-Ni60), no ash

content resulted in the temperature-programmed oxidation. This could be an evident that after second step chlorination at 1200 °C, nickel substances are removed from the final material.

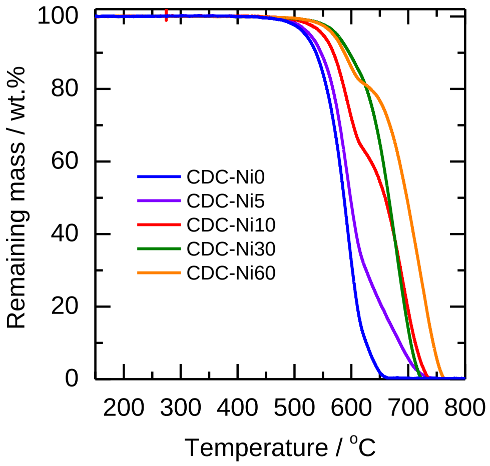

Figure S4: TPO curves of materials oxidized at oxygen atmosphere.

References

1. Schmirler, M.; Glenk, F.; Etzold, B. J. M. *Carbon* **2011**, *49*, 3679–3686. doi:10.1016/j.carbon.2011.05.003

2. Ariyanto, T.; Dyatkin, B.; Zhang, G.; Kern, A.; Gogotsi, Y.; Etzold, B. J. M. *Microporous Mesoporous Mater.* **2015**, *218*, 130–136. doi:10.1016/j.micromeso.2015.07.007
